# Hyb4mC: a hybrid DNA2vec-based model for DNA N4-methylcytosine sites prediction

**DOI:** 10.1186/s12859-022-04789-6

**Published:** 2022-06-29

**Authors:** Ying Liang, Yanan Wu, Zequn Zhang, Niannian Liu, Jun Peng, Jianjun Tang

**Affiliations:** College of Computer and Information Engineering, Jiangxi Agricultural University, Nanchang, China

**Keywords:** DNA N4-methylcytosine, Site identification, DNA2vec, Capsule Neural Network, Text Convolutional Neural Network

## Abstract

**Background:**

DNA N4-methylcytosine is part of the restrictive modification system, which works by regulating some biological processes, for example, the initiation of DNA replication, mismatch repair and inactivation of transposon. However, using experimental methods to detect 4mC sites is time-consuming and expensive. Besides, considering the huge differences in the number of 4mC samples among different species, it is challenging to achieve a robust multi-species 4mC site prediction performance. Hence, it is of great significance to develop effective computational tools to identify 4mC sites.

**Results:**

This work proposes a flexible deep learning-based framework to predict 4mC sites, called Hyb4mC. Hyb4mC adopts the DNA2vec method for sequence embedding, which captures more efficient and comprehensive information compared with the sequence-based feature method. Then, two different subnets are used for further analysis: Hyb_Caps and Hyb_Conv. Hyb_Caps is composed of a capsule neural network and can generalize from fewer samples. Hyb_Conv combines the attention mechanism with a text convolutional neural network for further feature learning.

**Conclusions:**

Extensive benchmark tests have shown that Hyb4mC can significantly enhance the performance of predicting 4mC sites compared with the recently proposed methods.

## Background

DNA methylation is a chemical modification of DNA, which influences the genetic performance while keeping the DNA sequence unchanged. Based on the basic genetic sequence, gene activity differs in differentially methylated regions (DMRs) [1]. Many studies have shown that DNA methylation influences gene expression by regulating DNA replication, changing the chromatin structure and the way DNA interacts with proteins [2]. DNA methylation represents an important regulator of gene transcription; hence, its biological function and mechanism have always attracted the interest of researchers [3].

The most common forms of DNA methylation are 5mC, 6mA and 4mC [4]. The differences between them are illustrated in descriptive images in Additional file 1: Fig. S1. The modification of 5mC and 6mA is not only important in prokaryotic genomes, but it also widely exists in high eukaryotic genomes, which have been extensively studied [5, 6]. Another important epigenetic modification is 4mC, which has been reported to mainly occur in prokaryotic genomes. In 4mC modification, which is catalyzed by the DNA methyltransferase, a methyl group is covalently bonded to the 4th carbon position of the cytosine in the genomic CpG dinucleotide.

The modification of 4mC contributes to our further understanding of epigenetic mechanisms. However, compared with the extensive research performed on 5mC [7] and 6mA modifications [8], we are still far enough from a deep understanding of the 4mC modification function. DNA 4mC has been confirmed to be involved in the correction and regulation of the errors in DNA replication [4], cell cycle control [9] and protection of host DNA from degradation [10]. Hence, detecting the distribution of 4mC sites in the genome is critical for further research regarding its biological function. However, our knowledge of restrictive modification systems is still insufficient [11], and our lack of knowledge about 4mC methyltransferases or restriction enzymes makes it difficult to detect the genome-wide location of 4mC.

High-throughput sequencing has revolutionized the field of epigenetics [11, 12]. Single molecule real-time (SMRT) sequencing [13] and 4mC-Tet-assisted bisulfite sequencing (4mC-TAB-seq) have been developed for 4mC sites identification [14]. The SMRT technology can directly detect 4mC sites without the need for reference genomes [13]. However, it still cannot be considered as an ideal method to handle thousands of samples in the R-M system [14]. For bacterial species with an existing reference genome, 4mC-Tet-assisted bisulfate sequencing can perform quick genome-wide detection of the 4mC sites in a cost-effective way [14].

Due to the cost and time consumption of the experimental methods, several machine learning and deep learning methods have been proposed for 4mC sites prediction (Additional file 1: Table S1), such that they represent a supplement to the biological experiments.

By encoding DNA sequences according to the nucleotide chemistry and frequency, the iDNA4mC tool enables the identification of 4mC sites using the support vector machine (SVM) algorithm [15]. Due to the high false positives and false negatives, which may increase the verification cost of the biological experiment, four sequence-based encoding schemes were integrated in the 4mcPred-SVM tool to enhance the feature extraction capabilities [16]; this enhanced the prediction performance on each species. Although several predictors were developed for the prediction of 4mC sites, it has been difficult to achieve equal performance when applied to genome-scale prediction [17]. To this end, eight feature descriptors were further considered in the 4mcPred-IFL tool, and iterative feature representation was introduced to improve the classification ability of the SVM algorithm [17].

Deep learning extracts the distributed feature representation of the data by transforming low-level features into more abstract high-level features or representation attribute categories. In the last few years, it has been widely applied in bioinformatics research [18–20]. The 4mCCNN tool uses one-hot encoding matrix and convolutional neural network (CNN) to detect 4mC sites [21]. However, due to the small deep learning architecture and datasets used in the algorithm, the learning ability of 4mCCNN could not be further expanded [22]. To further improve the prediction performance, the DNA4mC-LIP tool integrates six existing classical predictors [15–17, 23–25]. DNA4mC-LIP explores the best weights and then assigns them to each predictor through a linear iterative strategy. A comparison study on independent test datasets showed that DNA4mC-LIP achieved an enhanced performance. Based on the sequence encoding schemes used by previous researchers, the Deep4mC tool discusses and selects four more representative schemes to construct the input of the CNN [26]. To further extend the deep learning framework, a bootstrapping method was used for species with a small number of samples. Compared with the existing approaches, Deep4mC achieved better performance.

**Fig. 1:**
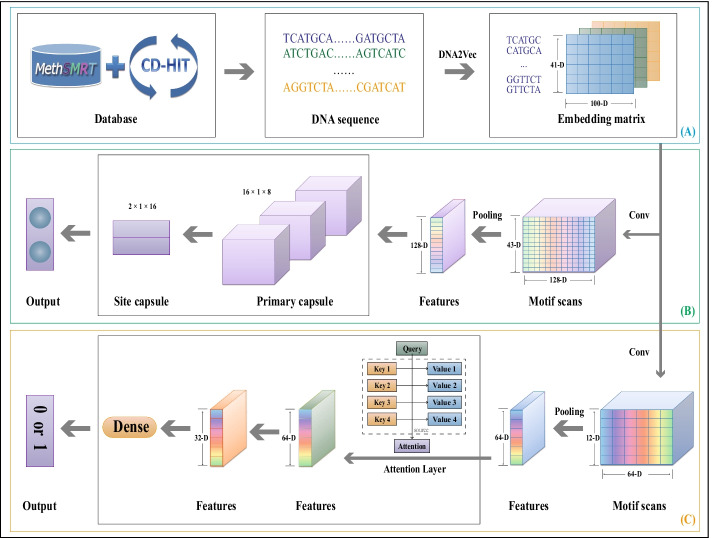
The framework of Hyb4mC. **A** The process of Hyb4mC dataset construction and sequence embedding. **B** The architecture of the Hyb_Caps subnet, which is suitable for predicting 4mC sites in small sample species, such as *E. coli*, *G. subterraneus* and *G. pickeringii*. **C** The architecture of the Hyb_Conv subnet, which is suitable for predicting 4mC sites in large sample species, such as *C. elegans*, *D. melanogaster* and *A. thaliana*

With the gradual progress in the experimental 4mC site identification methods, the scale of the available 4mC sample size of multiple species has been greatly expanded. As a result, there is now a big difference in the sample size among different species. The number of samples has a significant impact on the predictor performance. Although many prediction tools already exist to enable the identification of 4mC sites, the large variability in the sample size of some species makes many prediction tools unsuitable for today’s prediction tasks. To further understand the function of 4mC modification, a suitable method for 4mC site prediction of species with different sample sizes is necessary. To this end, we propose a flexible hybrid DNA2vec-based framework, called Hyb4mC. The basic structure diagram of the framework is shown in Fig. 1. Hyb4mC firstly uses the sequence embedding method based on DNA2vec [27] to improve the representative ability of feature descriptors. Then, two different subnets, Hyb_Caps and Hyb_Conv, are used to enhance the performance of 4mC site prediction in multiple species. Hyb_Caps is constructed using a capsule neural network, for the first time for this task, based on dynamic routing algorithm. Meanwhile, Hyb_Conv uses the attention mechanism to capture more critical features in order to make accurate predictions. Compared with existing available predictors on independent test datasets, Hyb4mC achieves better prediction performance.

## Results and discussion

In this section, we discuss the performance of our proposed framework Hyb4mC in detail. Similar to most previous researchers, we started the discussion based on six species: *C. elegans*, *D. melanogaster*, *A. thaliana*, *E. coli*, *G. subterraneus* and *G. pickeringii* [15–17, 21–26]. Most of these species are model organisms, we conduct research based on these species and hope to provide guidance for related or follow-up research. The intuitive distribution of the datasets is shown in Additional file 1: Table S2.

We explicitly construct species-specific models in the following studies by getting an overview of motif differences and similarities between different species. The improvement in the performance of Hyb4mC using the capsule neural network is illustrated by further analysis. At the same time, by visualizing the data distribution of Hyb_Caps and Hyb_Conv on the same species, the necessity of employing two sub-networks became clearer. The performance of Hyb4mC was evaluated using independent test datasets of Hyb_2021 and Li_2020, as well as comparisons with other state-of-the-art predictors; these results are significant because they demonstrate the improved prediction performance and robustness of Hyb4mC. Besides, based on the visualization of the similarity of motifs between different species enabled, cross-species validation was conducted to elucidate the link between the efficiency of knowledge transfer between species and their sequence motif similarity. Finally, further discussion and development of Hyb4mC’s limitations is conducted.

### Sequence analysis of conserved motif specificity

In order to accurately reveal the specific distribution of nucleotides around the 4mC/non-4mC sites among different species, Hyb_4mC analyzed the training datasets for each species using the pLogo generation tool [28]. Based on the visual sequence motif identification of the Hyb_2021 dataset, we analyzed the nucleotides that are significantly over-represented or under-represented at each position in the sequence for each species (Additional file 1: Fig. S2).

In *C. elegans*, guanine (G) and cytosine (C) were significantly enriched at positions +7 and +4, respectively, while thymine (T) and adenine (A) were significantly over-represented at most positions. The nucleotide distributions of *D. melanogaster*, *A. thaliana* and *E. coli* were similar in some regions, such that they all showed the enrichment of G in the $-1$, +1 to +3 region and the enrichment of C at the $-2$ position. However, compared with *D. melanogaster* and *A. thaliana*, A and T were significantly enriched only at a few positions in *E. coli*. In *G. subterraneus* and *G. pickeringii*, C and G were significantly over-represented at upstream ($-1, -2$ positions) and downstream positions (+1, +2, +6 positions) of the 4mC sites. Besides, in *G. subterraneus*, A was significantly enriched at the +5 position.

In addition, we analyzed the Li_2020 dataset, which was proposed by Li [22] (Additional file 1: Fig. S3 and Table S3). The distribution of nucleotides surrounding 4mC sites was species-specific, according to the sequence logos.

### Improving the predictive performance using the capsule neural network

**Fig. 2:**
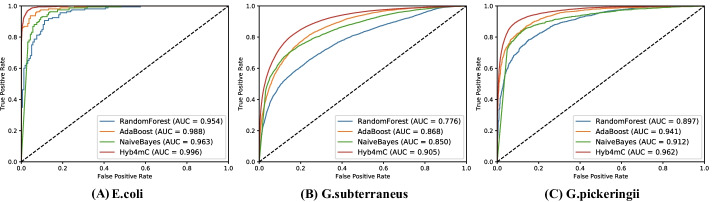
Performance comparison of the capsule neural network with classical ML-based classifiers on the independent test dataset of Hyb_2021

Since capsule neural networks could improve the generalization performance on few samples, we used it in our work to improve the prediction performance of species with insufficient sample size. To perform a fair comparison, the 41*100 feature matrix of sample sequence was used as the input to train the three classifiers of RandomForest [29], AdaBoost [30] and NaiveBayes [31]. By comparing the independent test datasets of three species, the capsule neural network was shown to have an improved performance. Based on the same feature extraction module, the AUC value was increased to 0.996, 0.905 and 0.962 in *E. coli*, *G. subterraneus* and *G. pickeringii*, respectively. The ROC curves are plotted in Fig. 2 (Additional file 1: Table S4, S5).

### Comparing the predictive performance using Hyb_Conv and Hyb_Caps

**Fig. 3:**
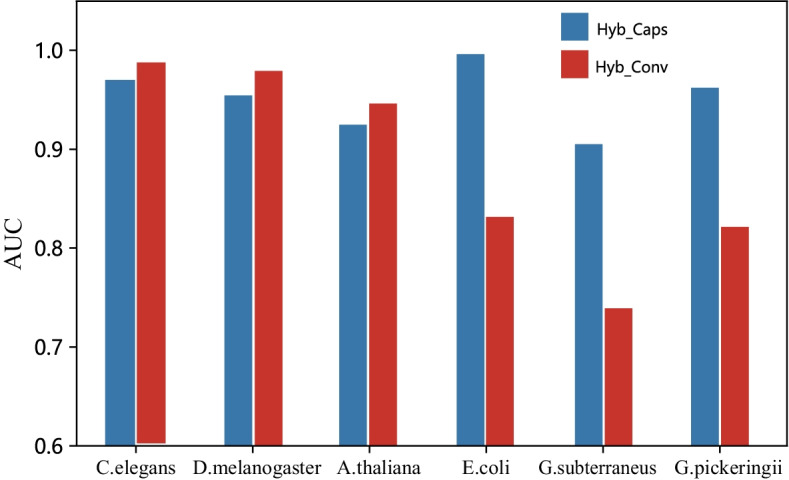
Performance comparison of Hyb_Conv and Hyb_Caps on six species from Hyb_2021

To show the difference between the two subnets, we compared the prediction performance of Hyb_Conv and Hyb_Caps on the same species. As shown in Fig. 3, on three species with large sample sizes (*C. elegans*, *D. melanogaster* and *A. thaliana*), Hyb_Conv improves prediction performance even further. Hyb_Caps significantly improves the prediction performance on three species with small sample sizes (*E. coli*, *G. subterraneus* and *G. pickeringii*), with an average increase of 19.8%.

**Fig. 4:**
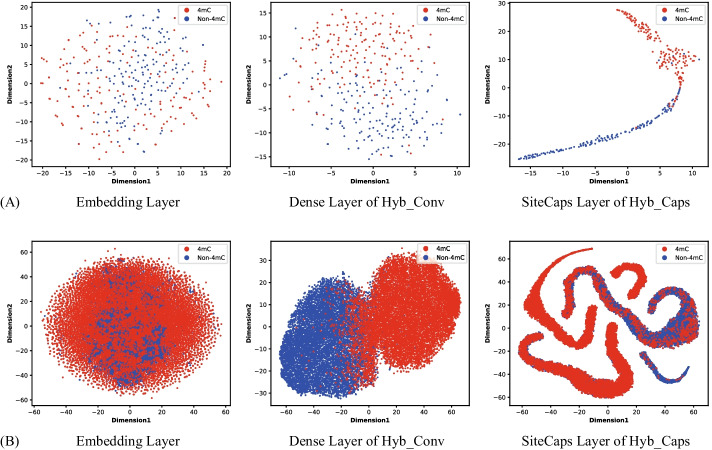
t-SNE plots of the embedding layer, dense layer of Hyb_Conv and SiteCaps layer of Hyb_Caps. **A** On *E. coli*, Hyb_Caps shows better classification performance. **B** On *C. elegans*, Hyb_Conv shows better classification performance

To further illustrate the classification performance of the two subnetworks, we used the t-distributed stochastic neighbor embedding (t-SNE) method to plot the state of data distribution at specific layers in the network. While t-SNE plots of the embedding layer described the original distribution state of the samples, t-SNE plots of the dense layer of Hyb_Conv and SiteCaps layer of Hyb_Caps were used to show the respective classification effects of the two subnetworks. Taking *E. coli* and *C. elegans* as examples, as shown in Fig. 4, 4mC and non-4mC sites contained samples were randomly distributed in the embedding layer. In Fig. 4A, the sample distribution of Hyb_Caps clearly has a stronger discrimination compared with Hyb_Conv. Meanwhile, Fig. 4B shows the sample distribution of Hyb_Conv is easier to distinguish on *C. elegans*, which has a large sample size. In addition, we also provided t-SNE plots of these three layers for other species discussed in this work (Additional file 1: Figs. S4 and S5).

### Performance on the Hyb_2021 and Li_2020 datasets

We performed performance evaluation tests on the Hyb_2021 and Li_2020 datasets, separately. We used our own dataset Hyb_2021 to train and test Hyb4mC. The individual AUC values for the six species of *C. elegans*, *D. melanogaster*, *A. thaliana*, *E. coli*, *G. subterraneus* and *G. pickeringii* reached 0.985, 0.979, 0.946, 0.996, 0.905 and 0.962, respectively.

For further evaluation, We used the Li_2020 dataset [22] and tested Hyb4mC on its independent test datasets of six species. The resulting individual AUC values for *C. elegans*, *D. melanogaster*, *A. thaliana*, *E. coli*, *G. subterraneus* and *G. pickeringii* reached 0.972, 0.980, 0.905, 0.964, 0.790 and 0.913, respectively. Although our test performance on *G. subterraneus* was not particularly satisfactory, the average AUC value of the other five species reached 0.947. For a more intuitive presentation, ROC curves are provided in Additional file 1: Fig. S6.

The prediction results on independent test datasets of six species from the Hyb_2021 and Li_2020 datasets showed the average AUC value for Hyb4mC on six species to be 0.962 (±0.001) and 0.920(±0.002), respectively. The individual confidence intervals for AUC values on the six species datasets of Hyb_2021 and Li_2020 are shown in Table 1. Hyb_4mC showed robustness on individual predictions for the six species.

**Table 1:** Confidence intervals for AUC values on six species datasets of Hyb_2021 and Li_2020

Species	AUC	
	Hyb_2021	Li_2020
*C. elegans*	0.985 ± 0.005	0.972 ± 0.004
*D. melanogaster*	0.979 ± 0.003	0.980 ± 0.003
*A. thaliana*	0.946 ± 0.005	0.905 ± 0.004
*E. coli*	0.996 ± 0.001	0.964 ± 0.005
*G. subterraneus*	0.905 ± 0.006	0.790 ± 0.008
*G. pickeringii*	0.962 ± 0.003	0.913 ± 0.003

### Performance comparison with the existing methods on the Hyb_2021 dataset

**Fig. 5:**
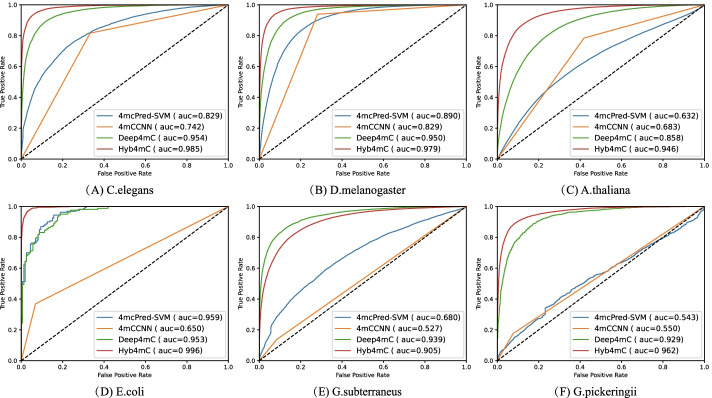
Comparison analysis of Hyb4mC with other methods in view of the AUC values on the independent test dataset of multiple species

**Fig. 6:**
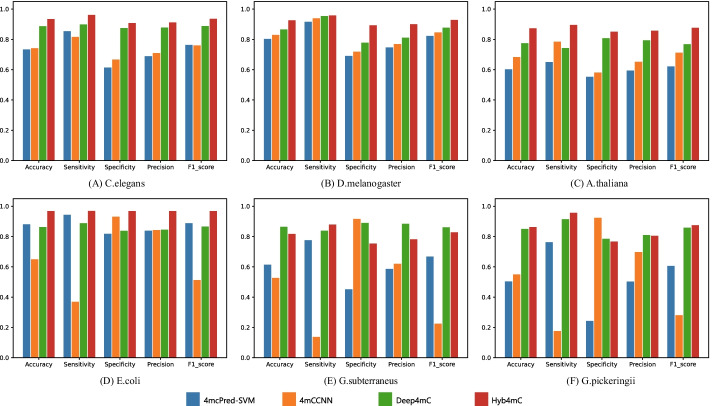
Performance comparison of Hyb4mC with other methods on the independent test dataset of six species

Many prediction methods have been developed to predict 4mC sites in the above-mentioned six species. While most methods provided a public web server instead of the source code, many of them were not accessible. We found the tools of 4mcPred-SVM [16], 4mCCNN [21] and Deep4mC [26] to be available. We used the Hyb_2021 independent test dataset to test the performance of Hyb4mC. For a fair comparison, the same independent test dataset was submitted to the above-mentioned web servers. Then, we downloaded the prediction results. Thus, we analyzed the performance difference between Hyb4mC and previous prediction methods, the predicted performance comparison is shown in Figs. 5 and 6.

As shown in Fig. 5, Hyb4mC achieved an enhanced performance in terms of the AUC value on five species, such that the AUC value was increased by 3.1%, 2.9%, 8.8%, 3.7% and 3.3% on *C. elegans*, *D. melanogaster*, *A. thaliana*, *E. coli* and *G. pickeringii*, respectively. Figure 6 shows the results of performance comparison in terms of other evaluation metrics. It can be observed that Hyb4mC achieved the best average performance on all five species except for *G. subterraneus*. Compared with the best performance that could be achieved by existing predictors, this indicated an increase of 4.6%, 6.4%, 2.8%, 3.4% and 4.9%, respectively (Additional file 1: Table S6).

### Comparison analysis with the existing methods on the Li_2020 dataset

In order to further investigate the performance of Hyb4mC, we compared its performance with that of three state-of-the-art predictors on the independent test datasets of Li_2020.

**Fig. 7:**
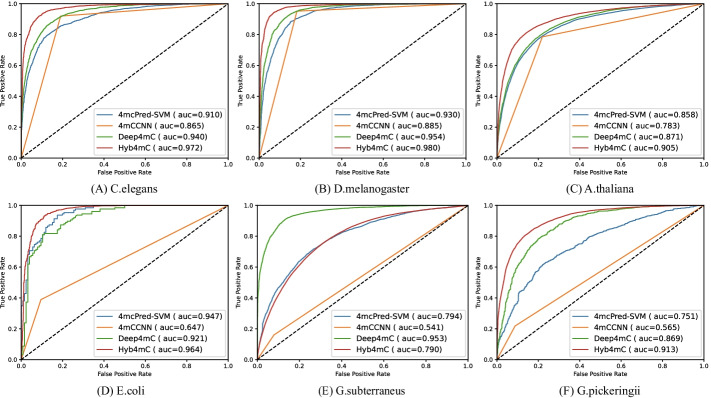
Comparison analysis between Hyb4mC and other methods in terms of AUC values on an independent test dataset

As illustrated in Fig. 7, compared with the state-of-the-art prediction methods, Hyb4mC achieved the highest AUC values in all species except for *G. subterraneus*. Compared with the optimal AUC achieved by previous predictors, Hyb4mC increased the AUC by 3.2%, 2.6%, 3.4%, 1.7% and 4.4%, respectively. For other evaluation metrics, Hyb4mC achieved average accuracy, sensitivity, specificity, precision and F1_score of 0.838, 0.932, 0.743, 0.797 and 0.856, respectively, across the six species. Compared with the best performance of existing predictors, Hyb4mC improved the accuracy, sensitivity and F1_score by 0.7%, 7.6% and 2.4%, respectively (Additional file 1: Fig. S7 and Table S7).

### Analysis of cross-species validation

The species category determines the number of 4mC sites that have been experimentally verified to a certain extent. The distribution of nucleotides around the 4mC site is species-specific. Exploring the relationship between this specific distribution and knowledge transfer between species enables us to further understand the relationships between different species defined by epigenetic states. We transfer the model parameters learned from data of another species to help train the new model. Based on this approach, we obtained six species-specific models by separately learning the training dataset of each species. These six species-specific models were applied to predict the 4mC sites of other species.

**Fig. 8:**
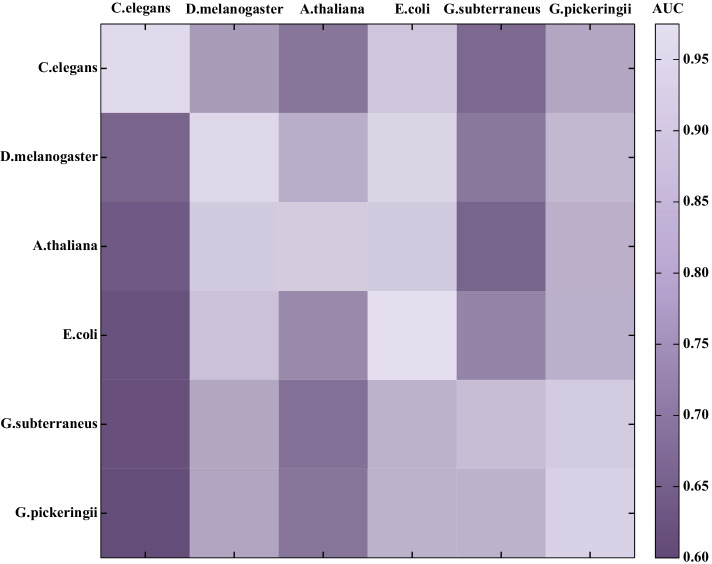
Heat map showing cross-species prediction performance

Figure 8 shows the prediction performance of the six species-specific models in form of a heat map. The cross-species AUC value corresponds to the color intensity of each square, and a change in the color from dark to light indicates an increase in the AUC value (Additional file 1: Table S8). As shown in Fig. 8, knowledge transfer among the three species of *D. melanogaster*, *A. thaliana* and *E. coli* achieved better performance. In addition, transfer learning between *G. subterraneus* and *G. pickeringii* also achieved a similar performance. Knowledge transfer among species shows a correlation with the specific distribution of nucleotides around the 4mC sites in different species. This distribution-specific information helps to explore relationships between different species defined by epigenetic states.

### Discussion and limitations

Instead of providing source code, most 4mC site prediction methods supplied a public web server, and many of them were inaccessible. Therefore, we compared with three tools 4mcPred-SVM [16], 4mCCNN [21] and Deep4mC [26] on Hyb_2021 dataset and Li_2020 dataset.

According to the comparisons on the Hyb_2021 dataset, The performance of 4mC-PredSVM and 4mCCNN on *G. subterraneus* and *G. pickeringii* was significantly inferior to the performance on other species. This may be due to the small deep learning architecture and dataset used in the algorithm. The learning ability of 4mC-PredSVM and 4mCCNN cannot be further extended, and it is difficult to adapt them to the prediction of some species, because their available data have been greatly expanded. However, compared with the other five species, 4mcPred-SVM obviously achieved a more enhanced performance on *E. coli*, possibly due to its small architecture, which is more suitable for the prediction of species with a small number of samples. In addition, the prediction performance of 4mCCNN on *E. coli*, *G. subterraneus* and *G. pickeringii* significantly decreased, which may be caused by the substantial increase in the amount of available data for the three species. Compared with other state-of-the-art predictors, Hyb4mC and Deep4mC achieved better robustness, and Hyb4mC achieved a better performance in multi-species 4mC site prediction.

As for comparisons on the Li_2020 dataset, Hyb4mC and Deep4mC showed more robust performance than other methods in the prediction of multi-species 4mC sites. It is worth mentioning that Deep4mC achieved better prediction performance on *G. subterraneus* compared with other predictors, which is probably due to its use of a bootstrapping method, extending its neural network framework. Besides, the performance of 4mcPred-SVM and 4mCCNN on some species was significantly better than other species with larger changes in the available data. For example, 4mCCNN managed to achieve a better performance on the three species of *C. elegans*, *D. melanogaster* and *A. thaliana*. The results indicated that Hyb4mC performed better not only on the Hyb_2021 dataset but also on the Li_2020 dataset provided by others.

Although our method achieves performance improvement over previous research methods, there are still some aspects that need further research. (1) In our work, DNA sequences with a modQV score $\ge$ 30 from the MethSMRT database can be considered as candidate positive samples, after the data processing process, Hyb_2021 datasets is constructed for prediction. However, the identification of the 4mC sites only by DNA sequences is bound to be limited, and further investigation of the relevant functional information of 4mC sites may be an effective supplement to the sequence information. (2) Currently, the embedding matrix is not updatable, if a larger training background is available in the future, each embedding vector may be able to capture more information. (3) Some motifs were discovered to have more significant effects on methylation levels [32], despite being able to extract the outputs of specified layers in the network (Additional file 2, Additional file 3), the traceability of important motifs in Hyb4mC remains difficult, finding available methods to backtrack to some important motifs may help to further refine our predictor.

## Conclusions

In this study, we proposed a prediction framework called Hyb4mC to predict the DNA 4mC sites. The advances in sequencing technology led to a huge gap in the number of experimentally verified samples among different species. In order to build an effective 4mC sites prediction model, we developed the Hyb4mC tool with two complementary subnetworks: Hyb_Caps and Hyb_Conv. The DNA2vec method was used for sequence embedding, with a 41*100 feature matrix containing more comprehensive and effective information compared with the sequence-based features. The convolution layer, maxpooling layer, PrimaryCaps layer and SiteCaps layer participated in Hyb_Caps. Meanwhile, the combination of text convolutional neural network and the attention mechanism in Hyb_Conv further improved the robustness of the 4mC site prediction performance across multiple species.

We used six species of *C. elegans*, *D. melanogaster*, *A. thaliana*, *E. coli*, *G. subterraneus* and *G. pickeringii*, and constructed an independent dataset for each species, called Hyb_2021. Compared with the current state-of-the-art methods, Hyb4mC significantly improved the performance of 4mC site identification. On the same independent test dataset of Hyb_2021, Hyb4mC increased the AUC of *C. elegans*, *D. melanogaster*, *A. thaliana*, *E. coli* and *G. pickeringii* to 0.972, 0.980, 0.905, 0.964 and 0.913, respectively. In addition, Hyb4mC also achieved an enhanced performance on the Li_2020 dataset.

This performance improvement can be attributed to the following factors: (1) The DNA2vec embedding vectors capture more efficient and comprehensive information than previous information features. (2) The capsule neural network achieves greater generalization with fewer samples. (3) Hyb4mC can flexibly adjust and use appropriate subnets according to different target prediction species to enhance the prediction performance.

In addition, many sequence-based features were used for 4mC site prediction by DNA sequences (like k-mer, MBE, RFHC, EIIP, etc.). The feature matrix obtained by the DNA2vec method effectively combines other complementary sequence features, which may further improve the prediction performance. However, the identification of the 4mC sites only by DNA sequences is bound to be limited. The information derived from DNA sequences is often limited to nucleotide sequence, frequency of occurrence, physicochemical information, etc., which have been widely used (Additional file 1: Table S1). However, it is difficult to satisfactorily improve the prediction effect. To the best of our knowledge, current research on the function of 4mC sites is not comprehensive. We consider that further investigation of the relevant functional information of 4mC sites can serve as an effective supplement to the sequence information, which may contribute to the development of more accurate and efficient predictors.

## Methods

### Datasets

Benchmark datasets from the MethSMRT database, proposed by Chen [15], have been extensively used. Since the MethSMRT database is being constantly updated, the benchmark datasets are small in comparison with the amount of data accessible. However, when trained on larger datasets, machine learning algorithms generally perform better and exhibit greater generalizability [33]. Hence, in this work, we constructed a new dataset, called Hyb_2021.

The verified 4mC sites of multiple species from the MethSMRT database were contained in Hyb_2021, including *C. elegans*, *D. melanogaster*, *A. thaliana*, *E. coli*, *G. subterraneus* and *G. pickeringii*. According to the description of methylation analysis technical [34, 35], the modification quality value (modQV) score shows that the IPD ratio is obviously different from the expected background, and a modQV score of 30 is the default threshold to regard a position as modified. Hyb_2021 selected the DNA sequences with a modQV score $\ge$ 30 from the MethSMRT database as candidate positive samples (4mC sites contained sequences).

Sequences in which SMRT does not detect central cytosine were regarded as candidate negative samples. Preliminary tests indicated that each sample was 41bp in length [15]. The CD-HIT program was utilized to filter similar sequences with a cutoff value of 0.7, since highly similar sequences may result in performance overestimation [36]. For the candidate positive samples processed with the CD-HIT program, we selected sequences with a modQV score $\ge$ 50 as a reliable independent test dataset, while the remaining sequences were used to construct the training datasets. The same numbers of negative samples were randomly chosen for a balanced dataset.

### Hyb4mC framework

Our proposed framework, Hyb4mC, used the sequence embedding module DNA2vec to learn higher-order features from the sequence, which has been proven to be an effective way to represent the features [37, 38]. For subsequent feature extraction, two subnets were developed: Hyb_Caps and Hyb_Conv.

#### Sequence representation

DNA2vec was proposed by Ng et al. [27] to calculate the distributed representation of variable-length k-mers in DNA. This method exploits genomic DNA sequences to learn the feature representation and converts each k-mer into a 100-D continuous vector space, inspired by word2vec, which uses a large corpus to train the model. Compared with the corresponding k-mer coding, the 100-D vector contains more comprehensive and effective information, which promotes the model ability to capture more comprehensive information.

**Fig. 9:**
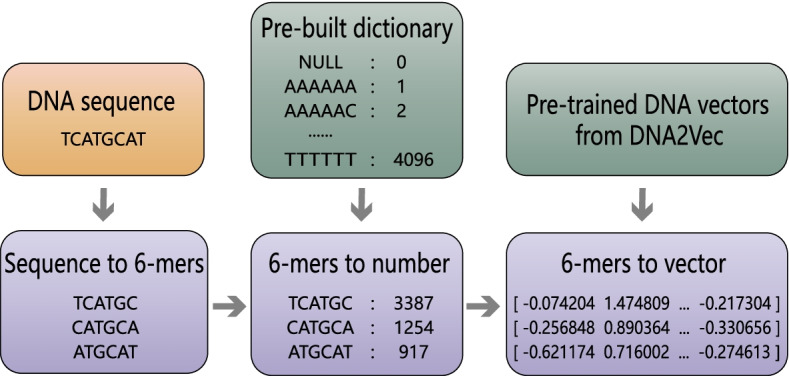
The sequence embedding algorithm

The positive 4mC and negative non-4mC sequences were divided into fixed-length k-mers by a sliding window. For example, “TCATGCAT” was divided into “TCATGC”, “CATGCA” and “ATGCAT”. The most commonly used encoding scheme is one-hot encoding, but as k increases, the dimension of the one-hot vector exponentially increases, potentially resulting in a dimensional explosion. Furthermore, one-hot encoding ignores the order between k-mers and assumes that the k-mers are independent of one another. Here, we employed sequence embedding approach instead of one-hot encoding. Considering the comprehensiveness of the information and the computational efficiency, k was set to 6. In our embedding approach, each 6-mer fragment corresponded to an index from 1 to 4096. In order to keep the number of 6-mer equal to the sequence length, we added “NNNNN” padding in front of the sequence, and the 6-mer that contained ‘N’ were indexed to 0. Every 6-mer was converted to a 100-D vector through the pre-trained DNA module, yielding an embedding matrix that represents the sequence information. The dimension of the matrix was 41*100. The process flow of sequence embedding is shown in Fig. 9.

#### Hyb_Caps

In the Hyb_Caps subnet, a capsule neural network was introduced to extract sequence features, and it is utilized here for the first time to predict 4mC sites. The capsule neural network was firstly proposed by Hinton et al. [39]. Capsules are not composed of neurons, but can be understood as a group of neurons in essence. The conventional capsule neural network consists of a PrimaryCaps layer and a SiteCaps layer, and a “dynamic routing” algorithm is used for propagation between the capsules [40]. Instead of the original translational invariance, the capsule neural network uses a new architecture that imitates the human visual system to obtain translational covariance, so that it needs less data to get more generalization under different perspectives.

To extract comprehensive features of the 41*100 matrix, a convolutional layer was used, with a total of 128 filters and a filter size of 5*100. A globalmaxpooling layer was used for downsampling after the convolutional layer, so that each DNA sequence corresponded to a 128-D vector.

In order to make reasonable use of all elements of the 128-D vector, and referring to the experience of previous researchers [41, 42], we used a total of 16 capsules, each with 8 elements, in the PrimaryCaps following the globalmaxpooling layer. The SiteCaps layer was used to store high-level vectors, which contained 2 capsules corresponding to our binary classification problem, each with 16 elements. The affine transformation was used to process the output of the PrimaryCaps layer, defined as Eq. 1:

1 $$\begin{aligned} {\hat{u}} =u\cdot h \end{aligned}$$

where *h* represents the affine transformation matrix, and *u* represents the output vector of the PrimaryCaps layer.

Finally, the two vectors in SiteCaps were modulated to predict the probability that the sample sequence contained 4mC sites.

It is worth mentioning that the dynamic routing algorithm was used for propagation from the PrimaryCaps layer to the SiteCaps layer, and based on literature, the number of routes T was set to 2. The dynamic routing algorithm pseudo code is shown in Algorithm 1. The example layer structure of the Capsule network and the process of dynamic routing is shown in Additional file 1: Fig. S8, $Cap_{i}$ is defined as the i-th capsule in the PrimaryCaps layer, $Cap_{j}$ is defined as the j-th capsule in the SiteCaps layer.
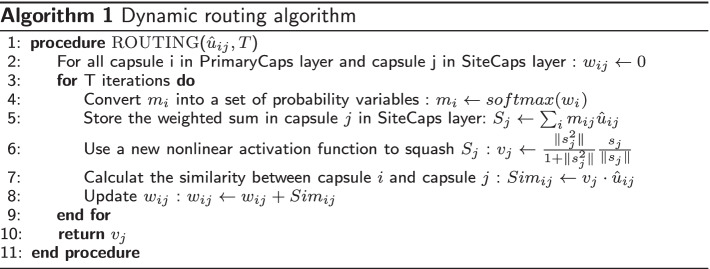


#### Hyb_Conv

In Hyb_Conv, a text convolutional neural network(TestCNN) was constructed with a convolutional layer and a maxpooling layer to further analyse the embedded 41*100 matrix. The amount of filters in the convolutional layer was set to 64, with a filter length of 60, a pooling length of 30 and a step size of 1. To prevent overfitting, we added a dropout layer with the value of 0.5.

The attention mechanism [43] has been widely used in various types of deep learning tasks [44–47]. The features extracted by the TextCNN were passed through an attention layer in the Hyb_Conv subnet. The more important the features, the higher the weights given through the attention mechanism, so that the model can capture more important features. The process of the attention mechanism is defined as Eqs. 2–4. The process diagram is shown in Additional file 1: Fig. S9. Furthermore, two dense layers with a sigmiod activation function were used to predict the 4mC sites.

2 $$\begin{aligned}&Sim_{i}=Query\cdot Key_{i} \end{aligned}$$

3 $$\begin{aligned}&W_{i}=Softmax(Sim_{i} )=\frac{e^{Sim_{i}} }{ {\textstyle \sum _{j=1}^{L_{x} }} e^{Sim_{j} } } \end{aligned}$$

4 $$\begin{aligned}&Attention={\textstyle \sum _{i=1}^{L_{x}}} W_{i}\cdot Value_{i} \end{aligned}$$

where *Query* represents the context vector, $Key_{i}$ represents the i-th feature, and $w_{i}$ represents the weight coefficient corresponding to $value_{i}$.

### Model training setup

Hyb4mC was implemented in Python 3.6 using Keras (2.1.6) with the backend of TensorFlow (1.12.0). In the Hyb_Caps subnet, a margin loss function was used, defined in Eq. 5. The training process used 10-fold cross-validation. The number of epochs was set to 30, and the batch size was set to 16. Meanwhile, we performed 10-fold cross-validation on the training dataset. In the Hyb_Conv subnet, the binary cross entropy was set as the loss function, defined in Eq. 6. The epoch was set to 90, and the batch size was set to 64. The Adam optimizer was used in both subnetworks.

5 $$\begin{aligned} L_{c} =T_{c} \max (0,m^{+}-\parallel V_{c}\parallel )^{2} +\lambda (1-T_{c})\max (0,\parallel V_{c}\parallel -m^{-} )^{2} \end{aligned}$$

where $m^+=0.9$, $m^-=0.1$, $\lambda =0.5$, and $T_c=1$ if category *c* is present.

6 $$\begin{aligned} Loss=-\frac{1}{N} {\textstyle \sum _{i=1}^{N}y_{i}\cdot \log (p(y_{i} ))+(1-y_{i}\cdot \log (1-p(y_{i} )) } \end{aligned}$$

where *y* represents binary tag 0 or 1, and *p*(*y*) represents the probability of belonging to the *y*.

### Evaluation metrics

The area under the ROC curve (AUC) was used to evaluate the performance of Hyb4mC. In addition, five widely used metrics were also used for performance evaluation [48–52], defined in Eqs. 7–11:

7 $$\begin{aligned}&{S_{n}} =\frac{TP}{{ TP}+ { FN}} \end{aligned}$$

8 $$\begin{aligned}&{S_{p}} =\frac{TN}{{ TN}+ { FP}} \end{aligned}$$

9 $$\begin{aligned}&\text {Precision} =\frac{TP}{{ TP}+ { FP}} \end{aligned}$$

10 $$\begin{aligned}&{A_{cc}} =\frac{{ TP}+ { TN}}{{ TP}+ { TN}+ { FP}+ { FN}} \end{aligned}$$

11 $$\begin{aligned}&{F_{1}}{score} ={ 1} - \frac{{ TP}+ { TN}}{{ 2}*{ TP}+ { FP}+ { FN}} \end{aligned}$$

where TP represents the number of true positives, TN represents the number of true negatives, FP represents the number of false positives, and FN represents the number of false negatives.

## Supplementary information

**Additional file 1:** Supplementary materials for Hyb4mC.

**Additional file 2:** An example of the matrix output by the Attention layer.

**Additional file 3:** An example of the matrix output by the Sitecaps layer.

## Data Availability

A code ocean capsule is available at: 10.24433/CO.7525832.v1. Source code (python) and the datasets are available at: https://github.com/YingLiangjxau/Hyb4mC.

## Abbreviations

4mC: N4-methylcytosine
DMRs: Differentially methylated regions
TextCNN: Text convolutional neural network
SMRT: Single molecule real-time
4mC-TAB-seq: 4mC-Tet-assisted bisulfite sequencing
SVM: Support vector machine
CNN: Convolutional neural network
AUC: The area under the ROC curve
TP: True positive
TN: True negative
FP: False positive
FN: False negative
