# Supplementary material for: Hyb4mC: a hybrid DNA2vec-based model for DNA N4-methylcytosine sites prediction
Source: BMC Bioinformatics. 2022 Jun 29;23:258. doi: 10.1186/s12859-022-04789-6 (PMC9241225; doi:10.1186/s12859-022-04789-6)
Supplement: Supplementary file 1 — Additional file 1: Supplementary materials for Hyb4mC. [file 12859_2022_4789_MOESM1_ESM.docx]

**Supplementary Figures**

**
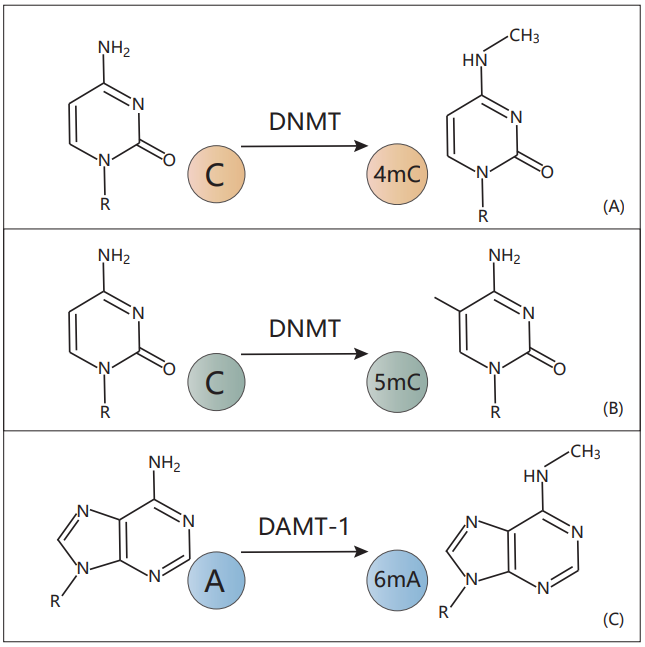
**

Figure S1. Three common DNA methylation. (A) 4mC, a methyl group is covalently bonded to the 4th carbon position of the cytosine. (B) 5mC, a methyl group is covalently bonded to the 5th carbon position of the cytosine. (C) 6mA, a methyl group is covalently bonded to the 6th carbon position of the Adenine.


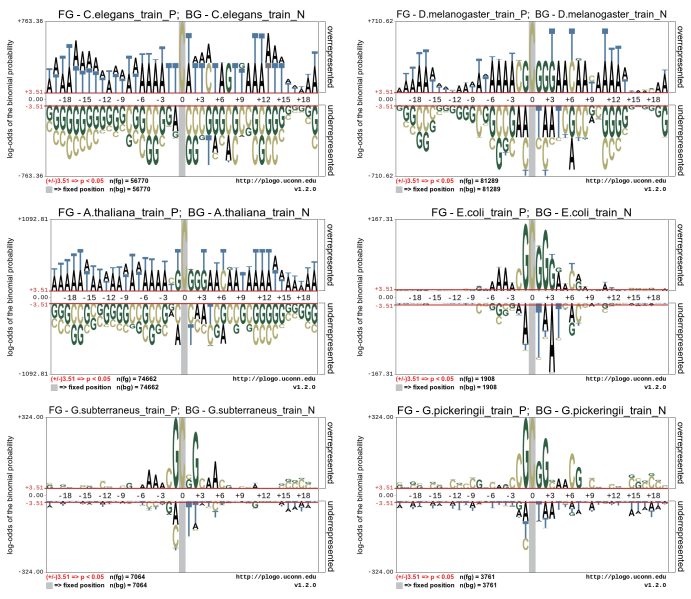


Figure S2. Sequence Logos between the 4mC sites and non-4mC sites of C.elegans, D.melanogaster, A.thaliana, E.coli, G.subterraneus and G.pickeringii of Hyb_2021 datasets. The red horizontal line corresponds to the significance threshold of 3.51, providing a convenient way to assess the statistical significance of the most significant residue at each position. (p < 0.05)

In C.elegans, guanine (G) and cytosine (C) were significantly enriched at positions +7 and +4, respectively, while thymine (T) and adenine (A) were significantly over-represented at most positions. The nucleotide distributions of D.melanogaster, A.thaliana and E.coli were similar in some regions, such that they all showed the enrichment of G in the -1, +1~+3 region and the enrichment of C at the -2 position. However, compared with D.melanogaster and A.thaliana, A and T were significantly enriched only at a few positions in E.coli. In G.subterraneus and G.pickeringii, C and G were significantly over-represented at upstream (-1, -2 positions) and downstream positions (+1, +2, +6 positions) of the 4mC sites. Besides, in G.subterraneus, A was significantly enriched at the +5 position.


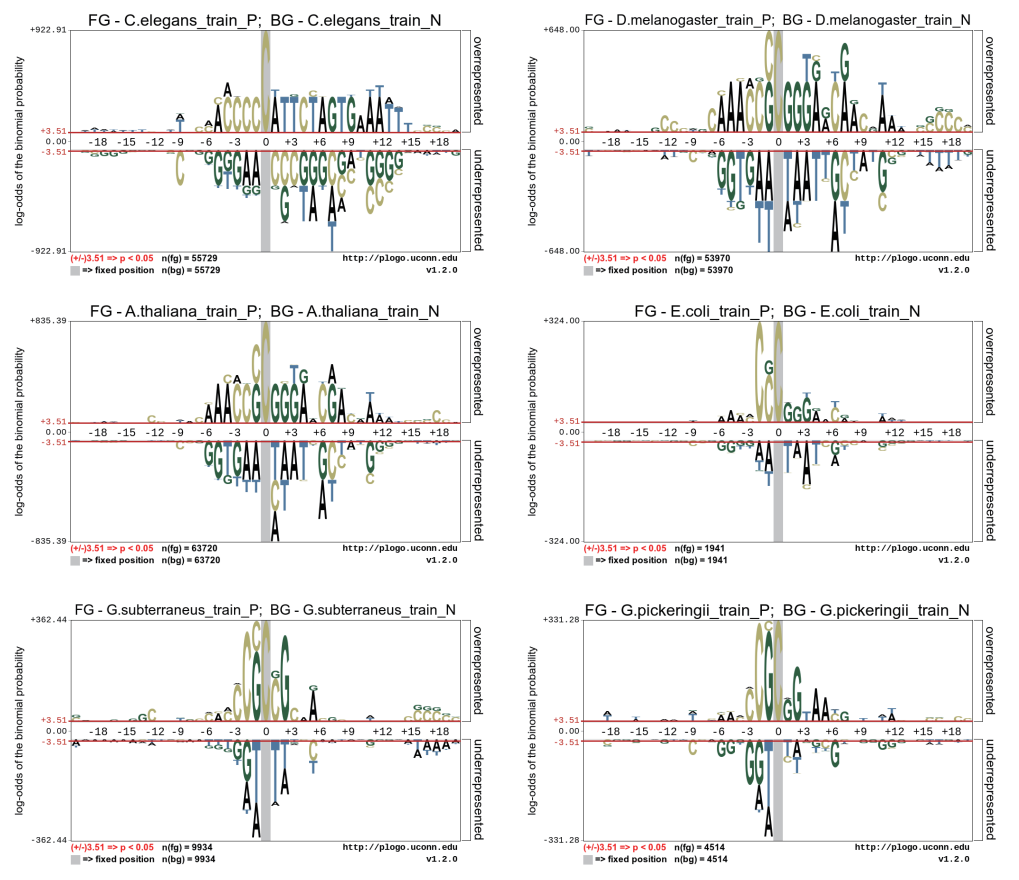
Figure S3. Sequence Logos between the 4mC sites and non-4mC sites of *C.elegans*, *D.melanogaster*, *A.thaliana*, *E.coli*, *G.subterraneus* and *G.pickeringii of Li_2020 datasets*. The red horizontal line corresponds to the significance threshold of 3.51, providing a convenient way to assess the statistical significance of the most significant residue at each position. (p < 0.05)

According to the Figure S3, in *C. elegans*, cytosine (C) was significantly enriched at -4~-1 region. The nucleotide distributions of *D.melanogaster*, *A.thaliana* and *E.coli* were similar in some regions, such that they all showed the enrichment of G in the -1, +1~+3 region and the enrichment of C at position -1, -2. However, compared with *D.melanogaster* and *A.thaliana*, A and T were significantly enriched only at a few positions in *E.coli*. In *G.subterraneus* and *G.pickeringii*, C and G were significantly overrepresented at upstream (-1, -2 , -3 positions) and downstream positions (+1, +2, +6 positions) of the 4mC sites. Besides, in *G.subterraneus*, A was significantly enriched at the +5 position.

**
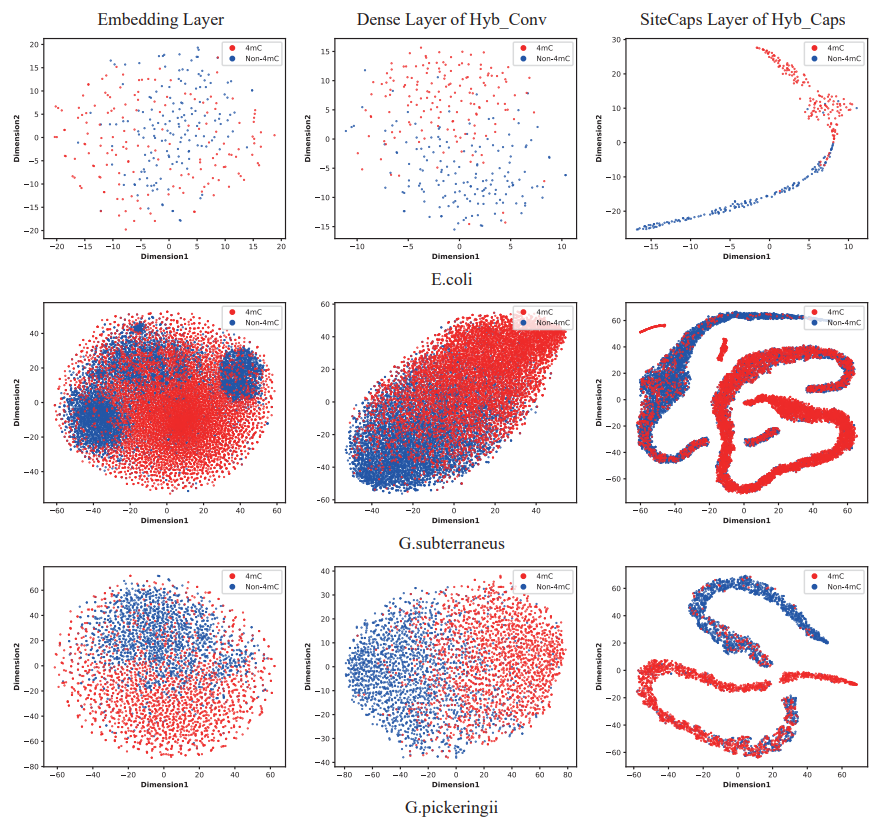
**

Figure S4. T-SNE plots of the Embedding layer, Dense layer and SiteCaps layer on three species (*E.coli*, *G.subterraneus* and *G.pickeringii*) in Hyb4mC. The T-SNE plots of the Embedding layer were used to display the initial distribution of datasets before Hyb_Conv or Hyb_Caps, and the T-SNE plots of the Dense layer and SiteCaps layer were used to display the classification by Hyb_Conv and Hyb_Caps, respectively.


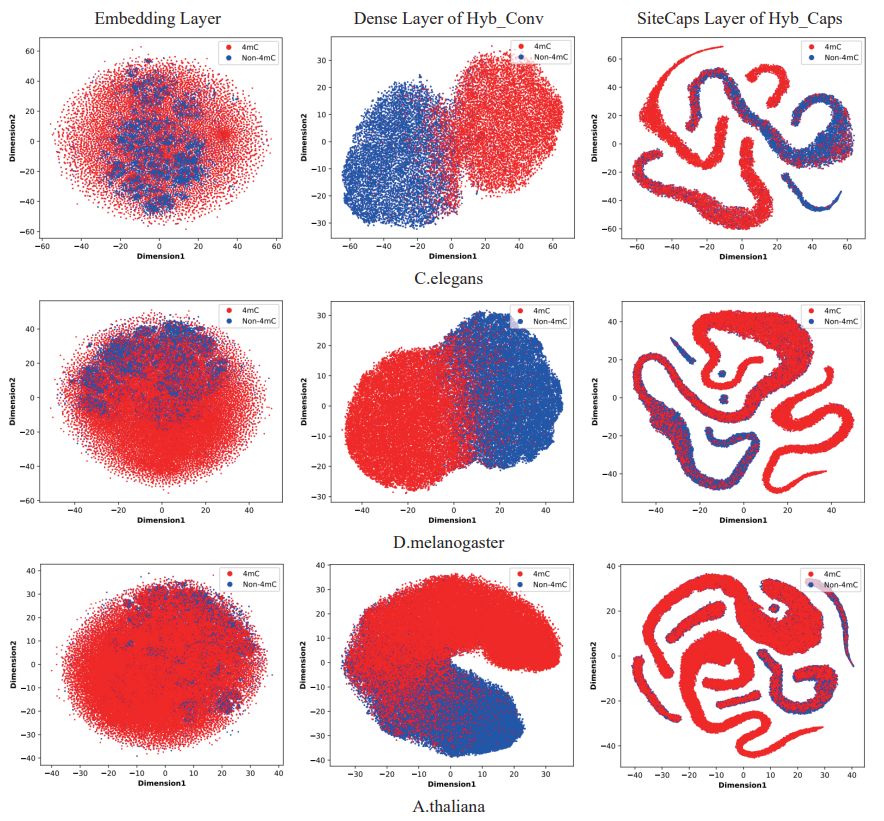


Figure S5. T-SNE plots of the Embedding layer, Dense layer and SiteCaps layer on three species (C.elegans, D.melanogaster and A.thaliana) in Hyb4mC. The T-SNE plots of the Embedding layer were used to display the initial distribution of datasets before Hyb_Conv or Hyb_Caps, and the T-SNE plots of the Dense layer and SiteCaps layer were used to display the classification by Hyb_Conv and Hyb_Caps, respectively.

**
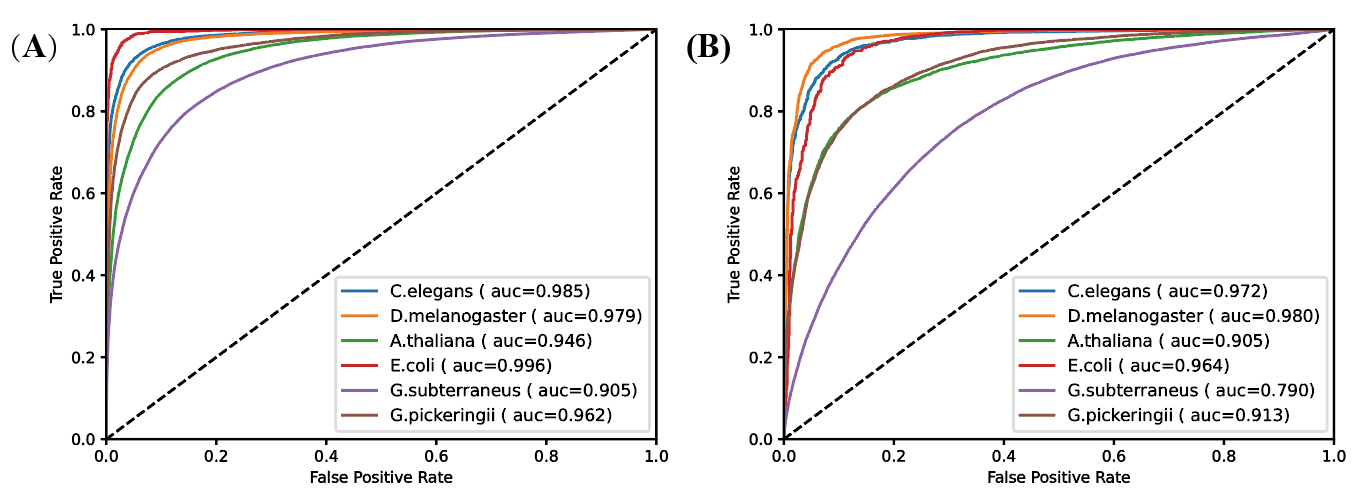
**

Figure S6. ROC curves of Hyb4mC on six species datasets. (A) ROC curve on Hyb_2021 independent test dataset. (B) ROC curve on Li_2020 independent test dataset.

**
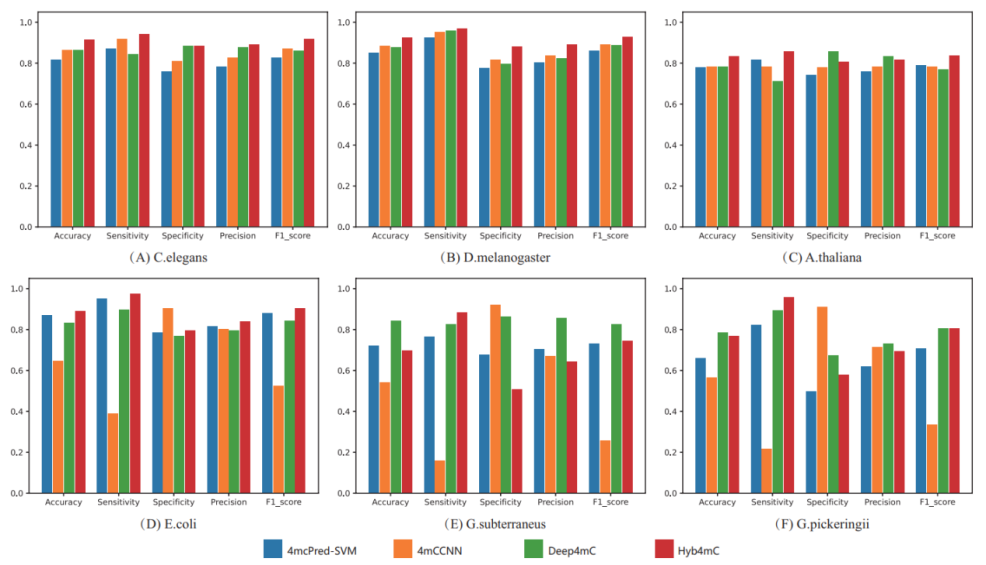
**

Figure S7. Comparison analysis of the five evaluation metrics of Hyb4mC with other methods on the independent test dataset.


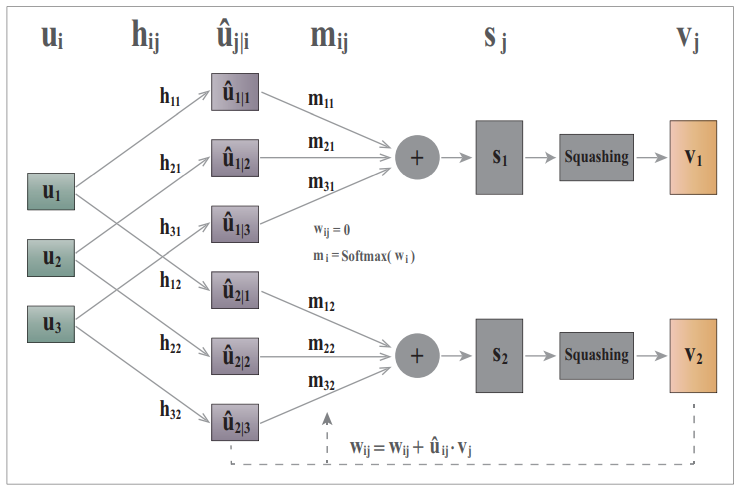


Figure S8. An example layer structure of the capsule network and the process of dynamic routing. $u_{i}$ is the output of ${Cap}_{i}$, $h_{ij}$ is the affine transformation matrix between ${Cap}_{i}$ and ${Cap}_{j}$, $\hat{u}_{ij}$ is the result of the affine transformation, $m_{ij}$ is the probability that ${Cap}_{i}$ will pass information to ${Cap}_{j}$, $s_{j}$ is the integration information of all capsules in the primary layer to ${Cap}_{j}$. $v_{j}$ is the output vector of ${Cap}_{j}$.


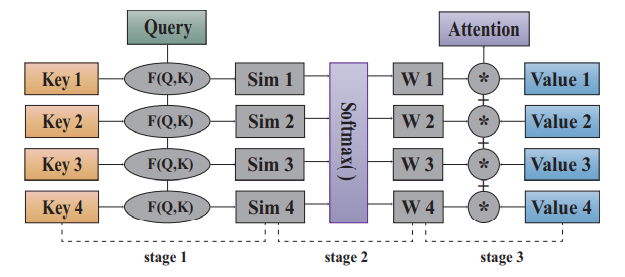


Figure S9. The process diagram of the attention mechanism.

**Supplementary Tables**

Table S1. Summary of existing 4mC Site Predictors.

| Predictor | Feature encoding scheme | Classifier | Average performance |
| --- | --- | --- | --- |
| IDNA4mc | NCP(Nucleotide chemical property)  NF(Nucleotide frequency) | SVM | ACC = 0.801  AUC = 0.884 |
| 4mCPred | NCP  EIIP(Electron-ion interaction potential)  PSTNP(Local position-specific trinucleotide difference) | SVM | ACC = 0.926 |
| 4mcPred-SVM | KF(K-mer frequency)  MBE(Mono-nucleotide binary encoding)  DBE(Dinucleotide binary encoding)  PSDNP(Local position-specific dinucleotide frequency) | SVM | AUC = 0.896 |
| Meta-4mCpred | MBE, KF, DBE, PSDNP, NCP  DPCP(dinucleotides physicochemical properties)  TPCP(trinucleotide physicochemical properties) | GB, ERT,  RF, SVM | ACC = 0.842  AUC = 0.904 |
| 4mcPred-IFL | KF+MBE, KF+DBE, NCP  PCPs(Physical-Chemical Properties)  PseDNC(Pseudo dinucleotide composition)  KNN(K-Nearest Neighbor)  PseEIIP(Electron-ion interaction pseudopotentials of trinucleotide)  MMI(Multivariate Mutual Information) | SVM | - |
| 4mCCNN | MBE | CNN | AUC = 0.926 |
| DNA4mC-LIP | - | integration of  six existing predictors | AUC = 0.941 |
| SOMM4mC | - | Markov | ACC = 0.881 |
| Deep4mC | MBE, EIIP, NCP  ENAC(enhanced nucleic acid composition) | CNN | AUC = 0.936 |
| DeepTorrent | NCP, EIIP, ENAC  CKSNAP(Composition of k-spaced nucleic acid pairs) | CNN | ACC = 0.870  AUC = 0.940 |

Table S2. Statistical summary of the Hyb_2021 dataset for the six different species.

| Species | Training datasets | |  | Test datasets | |
| --- | --- | --- | --- | --- | --- |
|  | Number of positive samples | Number of negative samples |  | Number of positive samples | Number of negative samples |
| *C.elegans* | 56770 | 56770 |  | 12147 | 12147 |
| *D.melanogaster* | 81289 | 81289 |  | 28000 | 28000 |
| *A.thaliana* | 74662 | 74662 |  | 50966 | 50966 |
| *E.coli* | 1908 | 1908 |  | 160 | 160 |
| *G.subterraneus* | 7064 | 7064 |  | 7813 | 7813 |
| *G.pickeringii* | 3761 | 3761 |  | 1926 | 1926 |

A dataset Hyb_2021 was constructed based on these six species, and training datasets for each species were constructed, containing 56770, 81289, 74662, 1908, 7064 and 3761 positive samples and a same number of negative samples, respectively. In addition, independent test datasets were constructed for the six species containing 12147, 28000, 50966, 160, 7813 and 1926 positive samples and a same number of negative samples, respectively. Each sample is a cytosine (C)-centered DNA sequence of 41bp in length (See the "Methods" section for details). Hyb_2021 contains more comprehensive samples of a high quality of each species, which can help to develop more robust and generalizable 4mC site prediction tools.

Table S3. Statistical summary of the Li_2020 dataset for the six different species.

| Species | Training datasets | |  | Test datasets | |
| --- | --- | --- | --- | --- | --- |
|  | Number of positive samples | Number of negative samples |  | Number of positive samples | Number of negative samples |
| *C.elegans* | 55729 | 55729 |  | 2667 | 2667 |
| *D.melanogaster* | 53970 | 53970 |  | 3684 | 3684 |
| *A.thaliana* | 63720 | 63720 |  | 11307 | 11307 |
| *E.coli* | 1941 | 1941 |  | 126 | 126 |
| *G.subterraneus* | 9934 | 9934 |  | 5263 | 5263 |
| *G.pickeringii* | 4514 | 4514 |  | 1210 | 1210 |

Table S4. Parameters setting of three classical classifiers.

| parameter settings | | |
| --- | --- | --- |
| RandomForest | AdaBoost | NaiveBayes |
| n_estimators = 500 max_leaf_nodes = 16 n_jobs = -1 | n_estimators = 200 algorithm = 'SAMME.R' learning_rate = 0.5 | - |

Table S5. Detail results of Capsule neural network for improving prediction performance.

| Species | Classifier | AUC |
| --- | --- | --- |
| *E.coli* | RandomForest | 0.954 |
|  | AdaBoost | 0.988 |
|  | NaiveBayes | 0.963 |
|  | Hyb4mC | **0.996** |
| *G.subterraneus* | RandomForest | 0.779 |
|  | AdaBoost | 0.868 |
|  | NaiveBayes | 0.850 |
|  | Hyb4mC | **0.905** |
| *G.pickeringii* | RandomForest | 0.898 |
|  | AdaBoost | 0.941 |
|  | NaiveBayes | 0.912 |
|  | Hyb4mC | **0.962** |

Table S6. Performance comparison with the existing methods on Hyb_2021 datasets.

|  | Predictor | Acc | Sn | Sp | Precision | F1_score |
| --- | --- | --- | --- | --- | --- | --- |
| *C.elegans* | 4mcPred-SVM | 0.734 | 0.854 | 0.614 | 0.689 | 0.763 |
|  | 4mCCNN | 0.742 | 0.816 | 0.667 | 0.710 | 0.760 |
|  | Deep4mC | 0.887 | 0.899 | 0.875 | 0.878 | 0.888 |
|  | Hyb4mC | **0.934** | **0.961** | **0.908** | **0.912** | **0.936** |
| *D.melanogaster* | 4mcPred-SVM | 0.803 | 0.916 | 0.691 | 0.747 | 0.823 |
|  | 4mCCNN | 0.829 | 0.939 | 0.719 | 0.769 | 0.846 |
|  | Deep4mC | 0.866 | 0.954 | 0.778 | 0.811 | 0.877 |
|  | Hyb4mC | **0.926** | **0.958** | **0.893** | **0.900** | **0.928** |
| *A.thaliana* | 4mcPred-SVM | 0.602 | 0.650 | 0.553 | 0.594 | 0.621 |
|  | 4mCCNN | 0.683 | 0.785 | 0.581 | 0.652 | 0.712 |
|  | Deep4mC | 0.775 | 0.743 | 0.808 | 0.794 | 0.768 |
|  | Hyb4mC | **0.873** | **0.895** | **0.851** | **0.857** | **0.876** |
| *E.coil* | 4mcPred-SVM | 0.881 | 0.944 | 0.818 | 0.839 | 0.888 |
|  | 4mCCNN | 0.650 | 0.369 | 0.931 | 0.843 | 0.513 |
|  | Deep4mC | 0.863 | 0.888 | 0.838 | 0.845 | 0.866 |
|  | Hyb4mC | **0.968** | **0.969** | **0.968** | **0.968** | **0.968** |
| *G.subterraneus* | 4mcPred-SVM | 0.614 | 0.776 | 0.452 | 0.586 | 0.668 |
|  | 4mCCNN | 0.527 | 0.137 | **0.916** | 0.620 | 0.225 |
|  | Deep4mC | **0.864** | 0.839 | 0.890 | **0.884** | **0.861** |
|  | Hyb4mC | 0.817 | **0.879** | 0.754 | 0.782 | 0.828 |
| *G.pickeringii* | 4mcPred-SVM | 0.504 | 0.763 | 0.243 | 0.503 | 0.606 |
|  | 4mCCNN | 0.550 | 0.176 | **0.924** | 0.698 | 0.281 |
|  | Deep4mC | 0.850 | 0.915 | 0.785 | **0.810** | 0.859 |
|  | Hyb4mC | **0.863** | **0.957** | 0.767 | 0.805 | **0.875** |

Table S7. Performance comparison with the existing methods on Li_2020 datasets.

|  | Predictor | Acc | Sn | Sp | Precision | F1_score |
| --- | --- | --- | --- | --- | --- | --- |
| *C.elegans* | 4mcPred-SVM | 0.816 | 0.873 | 0.760 | 0.783 | 0.826 |
|  | 4mcPred-IFL | 0.824 | 0.880 | 0.769 | 0.792 | 0.834 |
|  | 4mCCNN | 0.865 | 0.920 | 0.810 | 0.828 | 0.872 |
|  | Deep4mC | 0.864 | 0.844 | 0.884 | 0.879 | 0.861 |
|  | Hyb4mC | **0.915** | **0.944** | **0.886** | **0.893** | **0.918** |
| *D.melanogaster* | 4mcPred-SVM | 0.851 | 0.924 | 0.777 | 0.805 | 0.861 |
|  | 4mCCNN | 0.885 | 0.952 | 0.817 | 0.839 | 0.892 |
|  | Deep4mC | 0.878 | 0.960 | 0.796 | 0.825 | 0.887 |
|  | Hyb4mC | **0.926** | **0.970** | **0.883** | **0.892** | **0.929** |
| *A.thaliana* | 4mcPred-SVM | 0.781 | 0.819 | 0.743 | 0.761 | 0.789 |
|  | 4mCCNN | 0.783 | 0.785 | 0.781 | 0.782 | 0.783 |
|  | Deep4mC | 0.785 | 0.714 | **0.857** | **0.833** | 0.769 |
|  | Hyb4mC | **0.833** | **0.858** | 0.807 | 0.816 | **0.837** |
| *E.coil* | 4mcPred-SVM | 0.869 | 0.952 | 0.786 | 0.816 | 0.879 |
|  | 4mCCNN | 0.647 | 0.389 | **0.905** | 0.803 | 0.524 |
|  | Deep4mC | 0.833 | 0.897 | 0.770 | 0.796 | 0.843 |
|  | Hyb4mC | **0.890** | **0.975** | 0.796 | **0.841** | **0.903** |
| *G.subterraneus* | 4mcPred-SVM | 0.721 | 0.764 | 0.678 | 0.703 | 0.732 |
|  | 4mCCNN | 0.541 | 0.160 | 0.921 | 0.670 | 0.258 |
|  | Deep4mC | **0.844** | 0.826 | **0.862** | **0.857** | **0.826** |
|  | Hyb4mC | 0.697 | **0.885** | 0.508 | 0.644 | 0.746 |
| *G.pickeringii* | 4mcPred-SVM | 0.661 | 0.824 | 0.498 | 0.621 | 0.708 |
|  | 4mCCNN | 0.565 | 0.218 | **0.912** | 0.714 | 0.334 |
|  | Deep4mC | **0.784** | 0.894 | 0.674 | **0.733** | 0.805 |
|  | Hyb4mC | 0.769 | **0.959** | 0.578 | 0.695 | **0.806** |

Table S8. Detail results of cross-species validation.

|  | *C.elegans* | *D.melanogaster* | *A.thaliana* | *E.coli* | *G.subterraneus* | *G.pickeringii* |
| --- | --- | --- | --- | --- | --- | --- |
| *C.elegans* | **0.985** | 0.818 | 0.719 | 0.930 | 0.687 | 0.847 |
| *D.melanogaster* | 0.673 | **0.979** | 0.865 | 0.968 | 0.726 | 0.891 |
| *A.thaliana* | 0.647 | 0.942 | **0.946** | 0.942 | 0.675 | 0.869 |
| *E.coli* | 0.624 | 0.917 | 0.771 | **0.996** | 0.759 | 0.871 |
| *G.subterraneus* | 0.621 | 0.848 | 0.706 | 0.879 | **0.905** | 0.945 |
| *G.pickeringii* | 0.614 | 0.843 | 0.715 | 0.875 | 0.876 | **0.962** |

Table S9. AUC values of Hyb4mC based on different k-mer embedding.

| Species | AUC | | | | | |
| --- | --- | --- | --- | --- | --- | --- |
|  | 3-mer | 4-mer | 5-mer | 6-mer | 7-mer | 8-mer |
| *C.elegans* | 0.983 | 0.983 | 0.982 | **0.985** | 0.982 | 0.978 |
| *D.melanogaster* | 0.977 | 0.978 | 0.978 | **0.979** | 0.978 | 0.975 |
| *A.thaliana* | 0.945 | 0.940 | 0.940 | **0.946** | 0.938 | 0.933 |
| *E.coli* | 0.994 | 0.995 | 0.992 | **0.996** | 0.989 | 0.987 |
| *G.subterraneus* | 0.898 | 0.901 | **0.905** | **0.905** | 0.903 | 0.895 |
| *G.pickeringii* | 0.959 | 0.960 | 0.961 | **0.962** | 0.961 | 0.960 |
